# Development of an Antifungal Device Based on Oriental Mustard Flour to Prevent Fungal Growth and Aflatoxin B1 Production in Almonds

**DOI:** 10.3390/toxins14010005

**Published:** 2021-12-22

**Authors:** Tiago Melo Nazareth, Raquel Torrijos, Karla Paiva Bocate, Jordi Mañes, Fernando Bittencourt Luciano, Giuseppe Meca, Pilar Vila-Donat

**Affiliations:** 1 Department of Food Science and Toxicology, Faculty of Pharmacy, University of Valencia, Ave. Vicent Andrés Estellés s/n, 46100 Burjassot, Spain; raquel.torrijos@uv.es (R.T.); jorge.vmanes@uv.es (J.M.); giuseppe.meca@uv.es (G.M.); pilar.vila@uv.es (P.V.-D.); 2 School of Life Sciences, Pontificia Universidade Católica do Paraná, Rua Imaculada Conceiçao 1155, Curitiba 80125-901, Brazil; karla.bocate@pucpr.edu.br (K.P.B.); fernando.luciano@pucpr.br (F.B.L.)

**Keywords:** natural antimicrobials, fungi, mycotoxins, food safety, AITC, *Aspergillus flavus*, hydroxyethyl-cellulose-based device

## Abstract

The present study describes the manufacture of an antifungal device composed of oriental mustard flour and hydroxyethyl-cellulose (H-OMF) and evaluates its efficacity in inhibiting *Aspergillus flavus* growth and aflatoxin B1 (AFB_1_) production in almonds. Additionally, it compares the H-OMF with allyl isothiocyanate (AITC) and a freeze-dried extract of yellow mustard flour (YMF-E); such substances were previously described as antifungal. Minimum inhibitory concentration (MIC), Minimum fungicidal concentration (MFC), the H-OMF in vitro antifungal activity, and the residual fungal population, as well as the production of AFB_1_ in almonds were determined. AITC and YMF-E showed significant antifungal activity in vitro. Additionally, the in vitro activity of H-OMF avoided mycelial growth by applying 30 mg/L. Almonds treated with AITC (5.07, 10.13, and 20.26 mg/L) and H-OMF (2000 and 4000 mg/L) showed a reduction in the population of *A. flavus* and the production of AFB_1_ to values below the limit of detection. YMF-E showed effectiveness by in vitro methodologies (MIC and MFC) but did not show efficacy when applied in almonds. Our findings indicated that the hydroxyethyl-cellulose-based device containing oriental mustard flour might be utilised as a fumigant to increase the safety of almonds and could be extended to other cereals or dry fruits.

## 1. Introduction

Mycotoxins are secondary fungal metabolites produced mainly by the genera *Aspergillus*, *Penicillium*, and *Fusarium* [1,2,3]. Among the metabolites produced, aflatoxins (AFs) are produced by fungi of the genus *Aspergillus*, predominantly by two species, *Aspergillus flavus* and *Aspergillus parasiticus*, which can produce four main AFs, B_1_, B_2_, G_1_, and G_2_. According to International Cancer Research Agency (IARC), AFs are classified into group 1: carcinogens to humans. The AFB_1_ is the most toxic and can cause chronic liver damage, affect the immune system, growth, and malnutrition [4]. AFs are present in cereals, spices, and dry fruits such as almonds, peanuts, and oilseeds, contaminating directly pre-harvest and postharvest food [5].

Dry fruits such as almonds and nuts have beneficial effects, decreasing risk factors related to diabetes and cardiovascular diseases, with anti-inflammatory and antioxidant properties, and increasing bone health. Moreover, they are also rich in sugar and fat, mono- and polyunsaturated fats, and sterols [6,7,8]. Nevertheless, on the other hand, almonds are very susceptible to fungal growth and mycotoxin production. This fact occurs because of inadequate storage conditions, environmental issues (humidity and temperature), and high sugar content [9].

Several methods have been used to prevent fungal growth and mycotoxin production in foods. Among the strategies used are pre- and postharvest actions, which reduce the amount of contamination. Examples of pre-harvest actions are developing genetically modified cultivar lines, crop rotation, and changes in planting time, while post-harvesting methods include food drying, storage, and preservatives used [4].

Increasing interest in alternate preservation approaches for inactivating microbes and enzymes in foods has emerged from the need to ensure food safety while addressing such demands for nutrition and qualitative qualities [10]. Flavour, odour, colour, texture, and nutritional value are all critical quality characteristics. This growing demand for natural preservatives originating from plants, animals, or microorganisms has expanded the possibilities for their application [11]. Natural antimicrobials may be used alone or in combination with other innovative preservation technologies to enable the eradication of more conventional methods [12].

The use of essential oils has been investigated with great interest to the food industry due to their broad spectrum of activity, low tendency to induce resistance, and safety for consumption [13]. Plants belonging to the *Brassicaceae* family are rich in bioactive compounds, highlighting the glucosinolates, which in an enzymatic reaction give isothiocyanates (ITCs) [14]. *Sinapis alba* (yellow or white mustard) and *Brassica juncea* (brown or oriental mustard) are rich in glucosinolates and, in the presence of water, form ITCs’ aromatic compounds. In oriental mustard, myrosinase forms allyl isothiocyanate (AITC) from the main glucosinolate, sinigrin [15]. Concerning yellow mustard, the ITC formed is *p*-hydroxybenzyl isothiocyanate (ρ-HBIT) from the main glucosinolate sinalbin [16].

AITC is a volatile compound that is reported to exhibit numerous beneficial effects, including antimicrobial, anticarcinogenic, cardioprotective, and neuroprotective properties [17] and shows antifungal activity towards mycotoxigenic *Aspergillus* [18], *Penicillium*, [19], and *Fusarium* species [20]. Moreover, AITC is considered a GRAS (Generally Recognised as Safe) compound by the Food and Drug Administration (FDA), and the IARC has classified it as non-carcinogenic (class 3) [21].

Likewise, yellow mustard flour (YMF) has shown significant antimicrobial activity against food pathogens, such as bacteria and fungi [22,23].

In order to minimise the economic loss caused by fungi, the objectives of this work were, first, to evaluate the in vitro antifungal potential of the AITC and a freeze-dried yellow mustard flour extract (YMF-E). Then, it was to develop an antifungal device that contains hydroxyethyl-cellulose and oriental mustard flour (H-OMF) and determine its antifungal effect. Finally, it was to evaluate the efficacy of all treatments in avoiding the growth of *A. flavus* and preventing the production of AFB_1_ in almonds. These data may support a new strategy to preserve almonds and mitigate AFB_1_ contamination.

## 2. Results and Discussion

### 2.1. Determination of Minimum Inhibitory Concentration (MIC) and Minimum Fungicidal Concentration (MFC)

Using broth microdilution, *A. flavus* demonstrated sensitivity to AITC and YMF-E, as shown in Table 1. The MIC for AITC and YMF-E were 7.90 mg/L and 390 mg/L, respectively. The MFC of these compounds was previously compared with the results of MIC. AITC required a four-fold greater amount to have fungicidal effects. Regarding YMF, the quantity required was eight-fold greater than MIC. Thus, AITC was the compound that demonstrated more effectiveness against this toxigenic strain.

Many authors have studied the inhibition of fungal growth using natural compounds. Nielsen and Rios [24] tested the volatilisation of essential oils such as AITC against several fungi. Regarding *A. flavus*, the MIC was 3.5 mg/L in the gas phase. Other authors, such as Clemente et al. [25], also described the antifungal activity of AITC in a liquid medium. These authors identified that the MIC values for *A. flavus*, *A. ochraceus*, and *A. niger* were 6.25 mg/L, 6.25 mg/L, and 3.13 mg/L, respectively. However, MFC results were higher, ranging from 6.25 mg/L for *A. niger* and *A. flavus* strains and 25 mg/L for *A. ochraceus*.

Quiles et al. [26] reported MIC for several fungi strains using a concentrated extract of YMF non-autoclaved. The results ranged from 238.2 for *P. camemberti* to 15,000 mg/L for *A. flavus*, *A. parasiticus*, and *A. carbonarius*. In addition, this extract showed a fungicidal effect with MFC values ranging from 1875 mg/L against *P. nordicum*, *P. commune*, and *P. brevicompactum* to 15,000 mg/L towards *A. flavus*, *A. parasiticus*, and *A. carbonarius*.

YMF-E does not seem to have a substantial impact on *Aspergillus* spp., as does *Penicillium* spp., based on the findings reported by the other authors [11,27]. Conversely, we noticed that YMF-E had a lower MIC and MFC value in this study. Our findings, nonetheless, support previous studies since large doses are required to suppress *A. flavus* growth.

### 2.2. Oriental Mustard Flour (OMF) In Vitro Activity against A. flavus

Since the H-OMF device could not be diluted in a Potato Dextrose Broth (PDB), its antifungal activity was determined by measuring mycelial growth. Table 2 shows the in vitro antifungal effect of the H-OMF device against *A. flavus* during an incubation time of 7 days at 25 °C. Control samples reached a mean mycelial growth of 50 mm in diameter on the fifth day. Regardless of the incubation time, a dosage of 30 mg/L of H-OMF exhibited a fungicidal effect and inhibited visual mycelial growth. On day 3, a dosage of 25 mg/L of H-OMF reduced mycelial growth by 80%. On the fifth day, the same treatment showed a reduction by 50%. On the seventh day, this concentration provided a mycelial reduction by 26%. The dose of 12.5 mg/L of H-OMF significantly reduced mycelial growth on days 3 and 5; however, this dosage did not avoid the fungal growth. On the other hand, the concentration of 6.2 mg/L of H-OMF did not show an antifungal effect in the mycelial growth after 5 days of storage.

In general, only the concentration of 30 mg/L of H-OMF demonstrated a fungicidal effect; the other doses, such as 12.5 and 25 mg/L, only presented a fungistatic effect, which allowed the fungal growth during storage. Nevertheless, it should be highlighted that these concentrations also exhibited a significant difference regarding the control group. Therefore, these results suggest that H-OMF may inhibit the fungal growth in a dose-dependent manner, and concentrations higher than 30 mg/L of H-OMF might be necessary to achieve a fungicidal effect in complex matrices.

Manyes et al. [28] evaluated the capacity of AITC deposited inside a disc of sterile paper to avoid the micellar growth of *A. parasiticus* and *Penicillium expansum*. The authors demonstrated that the mycelial growth was not observed when AITC amounts greater than 50 mg were deposited in the Petri dishes’ centre inoculated with *P. expansum*. Concerning *A. parasiticus*, 25 mg was able to inhibit the mycelial growth completely. Saladino et al. [29] reported that OMF decreased the mycelial diameter growth of *A. parasiticus* by 48.2–60.4% when using 0.1 to 1 g of OMF incubated for 24 h. In our study, better results were found using a smaller amount of OMF, such as 0.030 g. This fact may be explained by the difference in the strains and the applied method by Saladino and colleagues.

### 2.3. Determination of the Fungal Population in Natural Almonds

Natural chemicals are increasingly being used to mitigate fungus development in foodstuffs. As shown in Table 3, on day 0, all treatments had significantly shown less fungal population than on days 7 and 15. All treatments of YMF-E tested did not inhibit the fungal growth when compared with the control group. However, 2000 and 4000 mg/L of H-OMF decreased the fungal population to levels below the limit of detection (LOD) (1.22 log CFU/g) on the 15th day. The same result was observed for the treatments with 5.07, 10.13, and 20.26 mg/L of AITC. Likewise, these concentrations decreased the fungal population to levels below the LOD on days 7 and 15. This fact may be explained because OMF in the presence of water was converted in AITC. However, this reaction did not occur with YMF-E. Concerning the residual fungal population, on day 0, there were no statistical differences among the control group and the treatments tested, which could suggest that antifungal compounds were slowly released over time.

Ground yellow mustard seeds and yellow mustard seeds have been paired with meats, especially as condiments for fermented sausages [30]. White or yellow mustard (*Sinapis alba*) and oriental or brown mustard (*Brassica juncea*) are known to contain a high concentration of glucosinolates, and these substances are cleaved in the presence of moisture by myrosinase to produce ITCs and a few other minor molecules such as thiocyanates and nitriles [26]. On the one hand, the predominant glucosinolate in yellow mustard is called sinalbin, which is cleaved by myrosinase to form ρ-HBIT. Although the antifungal effect of yellow mustard is not well understood, authors have attributed it to the synthesis of ITC compounds such as ρ-HBIT [30]. On the other hand, sinigrin is the major glucosinolate molecule found in oriental mustard seeds; these are nitrogen- and sulphur-containing metabolites that serve as AITC precursors. Similarly, AITC is enzymatically synthesized when sinigrin is hydrolysed by myrosinase in a humid environment [31]. Thus, OMF may be used as a natural supply of AITC that is gradually released into the headspace of silo systems [32].

Different mechanisms of action for ITCs’ antimicrobial activity have been suggested, including regulation of sulfhydryl enzymes, inhibition of ribonucleic acid (RNA) production, partial suppression of deoxyribonucleic acid (DNA) synthesis, and inhibition of protein synthesis through the ITCs’ molecule [33]. Although the antimicrobial mechanisms of ITCs are not fully understood, it is believed that their antimicrobial action is linked to their reactivity with proteins, which may disrupt in vivo biochemical processes. In other words, the carbon of the ITC molecule (R-N=C=S) is extremely electrophilic and interacts quickly with amines, thiols, and hydroxyls. Consequently, they may readily target the thiols and amines of the amino acid structure found in proteins. However, they primarily prefer to attack the sulfhydryl groups [34].

Authors also described that ITCs’ antibacterial action is a result of their amphiphilic nature. Although most authors agree that thiocyanate moiety is a critical component of ITCs’ antibacterial action, some authors have nevertheless described that ITCs exert their influence primarily through generating oxidative stress [19,24,35].

Our research group has demonstrated the antifungal capacity of AITC against a variety of fungi, such as *Aspergillus* [30] and *Penicillium* [28].

Lopes et al. [36] and Tracz et al. [21] showed the inhibition of fungal growth at levels of 2.5 and 500 µL/L of gaseous AITC (2.53 and 506.50 mg/L considering AITC density of 1.013 kg/m^3^). Nazareth et al. [37] reported a reduction of *A. parasiticus* CECT 2681 and *F. verticillioides* CECT 2983 at levels below the detection limit using a single treatment of 50 µL/L (50.65 mg/L) of AITC in corn kernels stored for 150 days. Our data show that a lower concentration of AITC was necessary to achieve comparable results. This difference can be justified because food matrices and fungal strains are not the same as reported previously [37]. Quiles et al. [38] demonstrated the growth inhibition of *P. verrucosum* using AITC; at the concentration of 500 µL/L (506.50 mg/L), the dispositive also reduced the fungal population to undetectable levels. Our study reported concentrations lower than those required in that study, which the differences in the applied methods could justify.

The antifungal activity of AITC was also evidenced by Suhr and Nielsen [39] using essential mustard oil with 99% of AITC on pieces of bread inoculated with 10^6^ spores/mL of *Penicillium roqueforti*, *Penicillium corylophilum*, and *A. flavus*. In that study, the inhibition of the fungi was observed at concentrations of 1 μL/L of AITC (99%). Quiles et al. [40] investigated the ability of AITC and OMF plus water to prevent the development of *A. parasiticus* in fresh pizza crust using active packaging devices for 30 days of storage. The growth of *A. parasiticus* was inhibited with AITC at 5 and 10 μL/L (5.07 and 10.13 mg/L, respectively) and OMF at 850 mg after 30 days. The treatment with 10.13 mg/L of AITC reduced the fungal population by more than 5 log CFU/g, and OMF at 850 mg reduced by 1.5 log CFU/g compared to the control group (8.99 log CFU/g).

Despite the differences between the studies mentioned above, this study proved that AITCs have intense antifungal activity against *A. flavus*, and natural compounds associated with novel devices, such as H-OMF, might be effective against mycotoxigenic strains in different types of food. Indeed, we propose that further research might be conducted to determine the fumigant potential of H-OMF in other dry fruits.

### 2.4. Aflatoxin Determination

To reproduce natural contamination of the almonds, *A. flavus* ISPA 8111 was used as an Afs’ producer. The results are shown in Table 4**,** and the samples were analysed from day 0 to day 15. As shown in Table 4, the treatments with H-OMF (2000 mg/L and 4000 mg/L) and AITC (5.07, 10.13, and 20.26 mg/L) reduced the production of AFB_1_, which could have resulted from the fungal growth inhibition. On the other hand, YMF-E (100, 160, and 200 g/L) had no effect inhibiting AFB_1_ production compared to the control group. Conversely, treatment with YMF-E significantly increased AFB_1_ production compared to the control group and over time. These results suggest that YMF-E should be avoided to prevent AFB_1_ production, or higher doses must be assayed.

This study may be considered the first in which a device based on hydroxyethyl-cellulose and OMF was applied to avoid the content of AFB_1_ in almonds. Nonetheless, the use of AITC to reduce the growth of the fungi and mycotoxin production has been studied previously by other authors. Nazareth et al. [20] evaluated the capacity of AITC to reduce the production of the mycotoxin by *A. parasiticus* and *Fusarium poae* in wheat flour. The results showed that the application of AITC in a concentration of 10 μL/L (10.13 mg/L) reduced the biosynthesis of mycotoxins entirely for 30 days. Tracz et al. [21] showed the capacity of AITC to reduce the production of mycotoxins in corn kernels in a concentration of 50, 100, and 500 μL/L (50.65, 101.30, and 506.50 mg/L, respectively), and all the treatments were capable of avoiding the production of mycotoxins. The data obtained in our study corroborate with these studies using AITC since the antimycotoxigenic effect was achieved applying concentrations lower than 20.26 mg/L.

Considering OMF’s effectiveness in reducing mycotoxin production, Quiles et al. [40] reported that pizza crust contaminated with *A. parasiticus*, when treated with 10 μL/L (10.13 mg/L) of AITC in filter paper and 850 mg of OMF, reduced the AFs’ (B_1_, B_2_, G_1_, and G_2_) production below the limits of quantification (≤LOQ). Hontanaya et al. (2015) evaluated the effectiveness of OMF in reducing the fungal growth of *A. parasiticus* and aflatoxin production in dry fruits (peanut, cashew, walnut, almond, hazelnut, and pistachio). The use of OMF reduced the aflatoxin production by 83.1–87.2%. Saladino et al. [29] used OMF (0.1, 0.5, and 1 g of flour) to derivate isothiocyanates and avoid aflatoxin production in Italian *piadina* contaminated with *A. parasiticus*. The reduction of aflatoxin production ranged from 60.5 to 89.3%. The same authors evaluated the use of isothiocyanates’ derivative from OMF and YMF by the water addition to reduce the formation of mycotoxins produced by *P. expansum*. The mycotoxin reduction ranged from 80 to 100%.

Recently, several authors have carried out intensive studies on the role of genes’ expression in the synthesis of AFs. Nevertheless, the regulation mechanism remains poorly recognized. Among the genes evaluated, aflR and aflS are strictly linked to the aflatoxin pathway, and their down-regulation, caused by environmental or nutritional factors, may lead the fungus to a suppressed aflatoxin production [41]. Within this frame of reference, Nazareth et al. [42] evaluated the AFB_1_ production and the transcriptional profile of *A. flavus*, applying sublethal dosages of AITC. All treatments increased the expression of genes involved in the synthesis of AFB_1_, although this mycotoxin has been substantially decreased because of the antifungal action. Alternatively, the findings indicated a widespread overexpression of the AFB_1_ gene cluster, which seems to be associated with the stressful condition induced by AITC activity. AITC also resulted in aberrant regulation of the examined genes, including those encoding critical transcription factors such as veA and laeA, which are also associated with the AFB_1_ production.

Overall, the device H-OMF reduced *A. flavus* growth and AFB_1_ production in the lab-scale system, demonstrating an important candidate for silo fumigation. Furthermore, this antifungal device could be scaled up to be tested in a large-scale experiment or a real silo trial due to its manufacturability. Therefore, it is also essential to note that further studies will be conducted to evaluate this antifungal device as a fumigator in 100-L scale and full-scale silos.

In a real-life situation, in order to maintain the overall quality during a longer storage time (more than 9 months), different physical barriers such as modified atmosphere packaging and refrigeration could be applied [43]. The use of low barrier packaging material combined with refrigeration on the storage conditions can maintain the quality of almonds for up to 12 months when stored at refrigeration temperature (2 °C) [43]. Therefore, due to the manufacturability of the antifungal device, it could be used in packaging based on permeable plastics or simply deposited on the bottom of storage containers.

## 3. Conclusions

The results obtained in this study showed the antifungal effectiveness of YMF-E, H-OMF, and AITC at in vitro assays towards *A. flavus* ISPA 8111, an important toxigenic fungus. Although the use of YMF-E did not show efficacy when used as a fumigant in almonds, AITC at 5.07, 10.13, and 20.26 mg/L and H-OMF at 2000 and 4000 mg/L demonstrated a remarkable ability to avoid the growth of *A. flavus* and AFB_1_ production. The AITC was slowly formed and released, inhibiting the fungal growth without contact with food. Thereby, the new device (H-OMF) based on hydroxyethyl-cellulose and oriental mustard flour could be used as a natural preservative agent to avoid fungal growth in almonds.

Comparing AITC and H-OMF, the results suggested that lower doses of AITC showed a similar antifungal and antimycotoxigenic effect as H-OMF on almonds. However, the results also demonstrated that all doses of H-OMF inhibited fungal growth and mycotoxin production, suggesting that the dose could be reduced. YFM-E was the least effective since treatments did not inhibit fungal growth and mycotoxin production.

Although AITC was more effective than H-OMF, essential oils’ compounds have a disadvantage, as they could modify the sensory characteristics of food products. On the contrary, the device (H-OMF) could gradually release the antifungal compound, avoiding this strong flavour and prolonging the safety of the stored products.

Therefore, further research should be conducted to determine the potential of AITC and H-OMF at lower concentrations, as well as the potential of YMF-E at higher dosages. Moreover, our results opened a plethora of possibilities, and further studies must be performed to determine the antifungal activity of H-OMF in different matrices such as cereals and other dry fruits. We also propose the application of this antifungal device against different types of processed almonds in association with physical barriers such as temperature and humidity control during different steps of the food chain. This association could prolong the shelf life of almonds and improve the antifungal potential of the device.

## 4. Materials and Methods

### 4.1. Chemicals

AFB_1_ standard solution (purity > 99%) was purchased from Sigma-Aldrich (St. Louis, MO, USA). The hydroxyethyl-cellulose was provided from Sigma-Aldrich (St. Louis, MO, USA). Methanol (99%) and formic acid (99%) used for liquid chromatography were HPLC grade and obtained from VWR Chemicals (Radnor, PA, USA). Ammonium formate was obtained from Sigma-Aldrich (St. Louis, MO, USA). Microbiological media such as PDB, potato dextrose agar (PDA), and Peptone Water were obtained from Liofilchem Products (Roseto Degli Abruzzi, Italy). YMF and oriental mustard flour (OMF) were provided by G.S. Dunn dry mustard millers (Hamilton, ON, Canada). AITC (95.1% purity) was purchased from Sigma-Aldrich (St. Louis, MO, USA).

### 4.2. Microorganism and Culture Conditions

The mycotoxigenic strain of *A. flavus* ISPA 8111 used in this study was obtained from the Institute of Food Production Science (ISPA, Bari, Italy). This microorganism was stored in sterile glycerol at −80 °C before use. Then the microorganism was grown in PDB at 25 °C, and, after growth, the PDA Petri dishes were inoculated in the dark.

### 4.3. Preparation of the Freeze-Dried Yellow Mustard Extract (YMF-E)

The method previously described by Quiles et al. [26] was used to extract water-soluble components with minor modifications. YMF (2 g) was homogenised in 25 mL of distilled water for 5 min at 7000 rpm using an Ultra Ika T18 basic Ultra-Turrax (Staufen, Germany). After centrifuging the extracts for 15 min at 5000× *g*, the supernatant was collected and deposited on polypropylene trays. Afterwards, the supernatant was freeze-dried for 72 h in a FreeZone 2.5 L Labconco (Kansas, MO, USA). The powder produced was kept at a temperature of 4 °C until its use in the antifungal activity test.

### 4.4. Manufacture of H-OMF Antifungal Device

**Figure 1:**
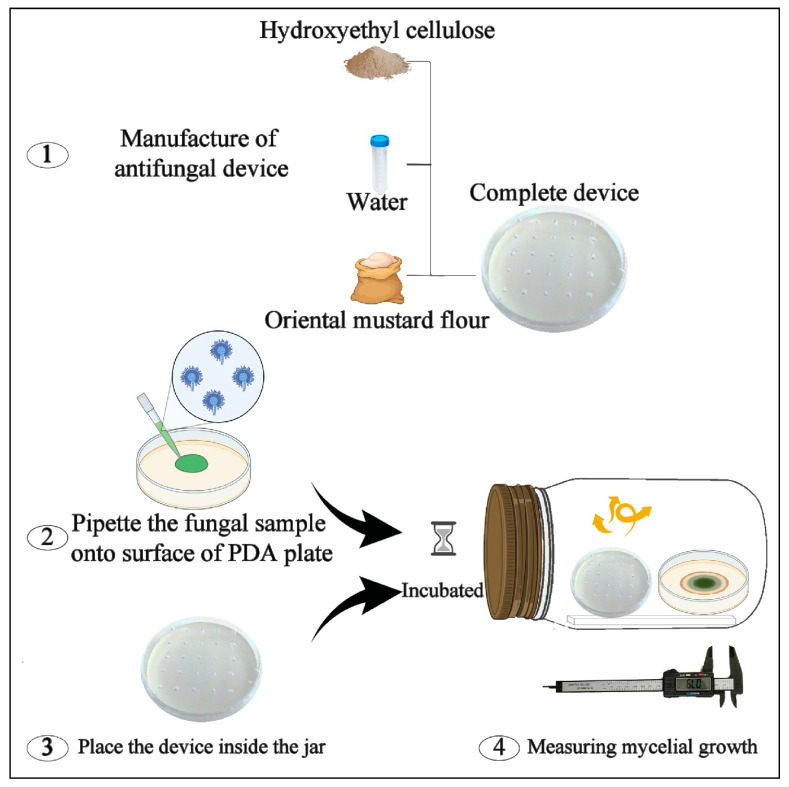
The small silo system used to determine the in vitro antifungal activity of mustard flour device based on hydroxyethyl-cellulose (H-OMF) against *Aspergillus flavus* ISPA 8111.

In a Petri dish, 1.5 g of hydroxyethyl-cellulose (gelling agent), 10 mL of water, and 2 or 4 g of OMF were combined to form the gel device. As illustrated in Figure 1, the lid of the Petri dish was previously punctured to allow the volatilisation of the AITC. The antifungal device was then inserted into the jars to study its antifungal effect.

### 4.5. Determination of the Minimum Inhibitory Concentration and the Minimum Fungicidal Concentration of AITC and YMF-E

The minimum inhibitory concentration (MIC) was determined by quadruplicate, using broth microdilution in a 96-well plate according to the protocol M38-A2 of the Clinical Laboratory Standard Institute, with adaptations [44]. The fungus was first grown on PDA and incubated for 7 days at 25 °C. The fungal suspension was prepared by harvesting the spores from the surface of the plates with peptone water 0.1%. After counting the number of spores using a Neubauer chamber, the inoculum was adjusted to 2 × 10^4^ spores/mL in a PDB medium (Liofilchem, Italy). Then, 100-μL aliquots of fungal suspension were added to each well. Different concentrations of AITC (ranging from 0.98 mg/L to 506.50 mg/L) and YMF-E (ranging from 90 to 100,000 mg/L) were added by microdilution. The microplate was filled to a final volume of 200 μL/well. The plate was incubated for 48 h at 25 °C before a visual reading was performed. Control groups were prepared with PDB and fungal suspension (positive control) and only PDB (negative control) containing 200 μL as the final volume.

The minimum fungicidal concentration (MFC) was determined after MIC determination according to the protocol described by Espinel-Ingroff et al. [45]. First, 10-μL aliquots of each well showing complete inhibition of fungal growth were withdrawn and cultured in PDA plates for 72 h at 25 °C. The negative control group was also inoculated in PDA plates and incubated for 72 h at 25 °C. The MFC was then established as the lowest dilution that yielded fewer than three colonies.

### 4.6. In Vitro Antifungal Activity of H-OMF against A. flavus ISPA 8111

Petri dishes (50-mm diameter) containing 6.2, 12.5, 25, and 30 mg of OMF were prepared to mix the flour with 10 mL of sterile distilled water (to improve the conversion of the glucosinolate sinigrin into AITC) and 1.5 g of hydroxyethyl-cellulose (a gelling agent). Simultaneously, Petri dishes (50-mm diameter) containing PDA were inoculated with 10 µL of *A. flavus* ISPA 8111 at 10^4^ spores/mL. After, the dishes were inserted into the jars without the lids, and then the jars were hermetically closed (Figure 1). The control group did not receive any treatment. Jars were kept at 25 °C for 7 days. Finally, on days 3, 5, and 7, the inhibitory effect of H-OMF was monitored by measuring the mycelia growth diameter on a scale of mm.

### 4.7. Microbiological Assay with Natural Almonds

**Figure 2:**
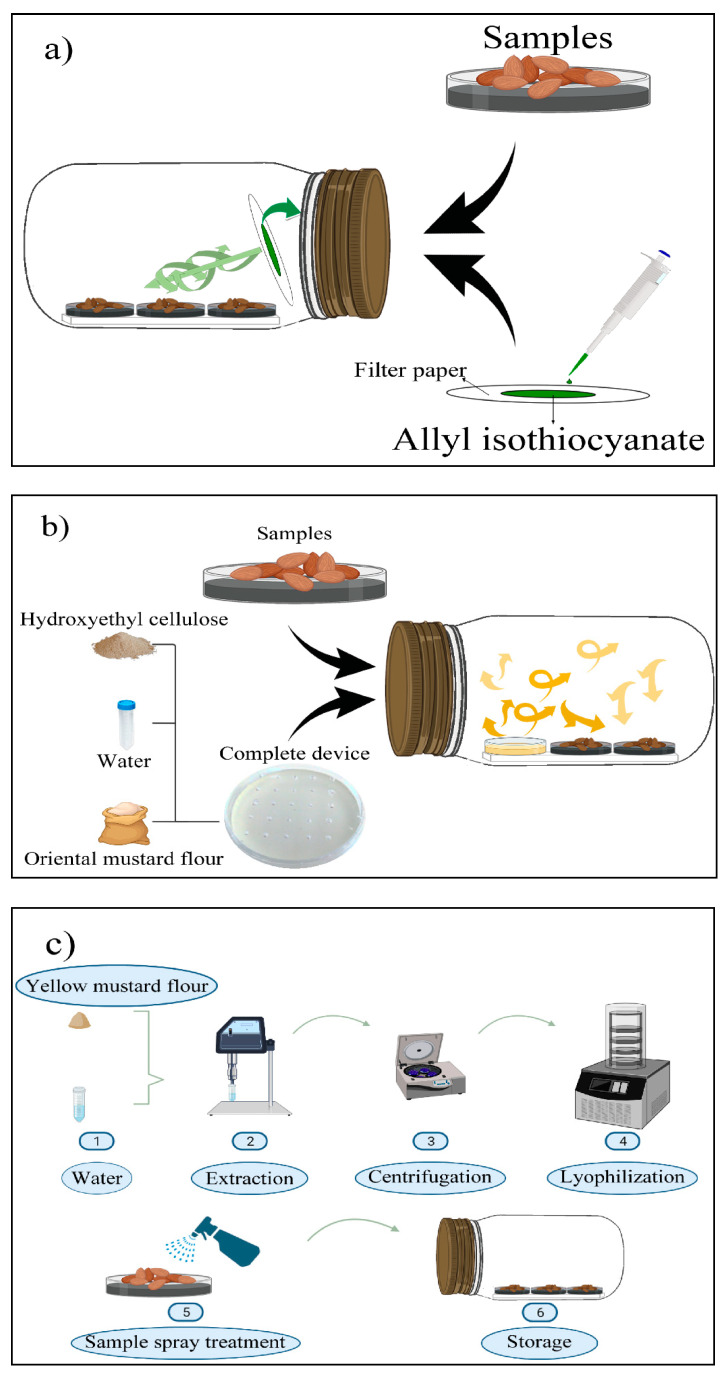
Silo system used to determine the volatile antifungal activity of: (**a**) allyl isothiocyanate (AITC); (**b**) the antifungal device based on hydroxyethyl-cellulose and oriental mustard flour (H-OMF); and (**c**) the spray of freeze-dried yellow mustard flour extract (YMF-E). Natural almonds were contaminated with *Aspergillus flavus* ISPA 8111 and stored for 15 days.

The natural almonds were autoclaved, dried at 28 °C for 24 h, contaminated with *A. flavus* ISPA 8111 in a concentration of 10^6^ spores/g, and then portioned in samples of 20 g. After 24 h of incubation, three different treatments were carried out:

- Filter papers (2.5 × 2.5 cm) containing AITC at 5.07, 10.13, and 20.26 mg/L (related to jar volume) were prepared and adhered to the lids of the jars (Figure 2a).
- H-OMF antifungal device was manufactured by mixing 2 and 4 g of OMF with 10 mL of sterile distilled water and 1.5 g of hydroxyethyl-cellulose. The device was then placed into the jars, reaching a final concentration of 2000 and 4000 mg/L (related to jar volume) (Figure 2b). These values represented 80- and 160-folds of the minimal dose to avoid the mycelial growth of *A. flavus* significantly.
- A spray of YMF was prepared with 0.5 (32 folds MFC), 0.8 (51.2 folds MFC), and 1 g (64 folds MFC) of YMF-E with 5 mL of sterile distilled water. The treatment was carried out by spraying the extract on the surface of almonds to reach final concentrations of 100, 160, and 200 g/L (Figure 2c).

The samples were placed in 50-mm Petri dishes and transferred to 1-L glass jars. Then, the jars were hermetically closed (Figure 2). Control samples did not receive treatments. Experiments were performed in triplicate for 15 days and kept at 25 °C. Finally, the almonds were microbiologically analysed on days 0, 7, and 15.

### 4.8. Determination of the Fungal Population in Natural Almonds

The numbers of moulds in almonds’ kernels during storage were analysed according to Hashemi and Raeisi (2018) [46].

After incubation, 20 g of each sample were transferred to a sterile plastic bag containing 180 mL of sterile peptone water 0.1% (Liofilchem, Italy) and homogenised with a stomacher (IUL, Barcelona, Spain) for 60 s. Serial dilutions of the suspensions were performed in sterile plastic tubes with 0.1% peptone water. After that, aliquots of 0.1 mL were plated on Petri dishes containing PDA (Liofilchem, Italy), and the plates were incubated at 25 ◦C for 7 days before microbial counting. The results were expressed in a log of colony-forming unit/g of almond (log CFU/g). All analyses were conducted in triplicate.

### 4.9. AFB_1_ Extraction

The AFB_1_ extraction was performed using the method previously described by Huang et al. [47] with some adaptations. First, samples were ground and homogenized and 5 g were taken in Falcon tubes of 50 mL with 25 mL of methanol. Then the extract was homogenised for 3 min by Ultra Ika T18 basic Ultraturrax (Staufen, Germany) at 13,500 RPM. Next, the extracts were centrifuged at 4800× *g* for 5 min at 4 °C, and the supernatant was transferred and evaporated using a Büchi Rotavapor R-200 (Postfach, Switzerland). Finally, the obtained residue was resuspended in 2 mL of methanol, filtered through a 0.22-µm syringe filter, transferred to a glass vial, and injected into an LC-MS/MS system.

### 4.10. LC-MS/MS Analysis

The liquid chromatography system was composed of an LC-20AD pump connected to a 3200QTRAP mass spectrometer (Applied Biosystems, Foster City, CA, USA) through an ESI interface operating in positive ion mode. The established stationary phase was a Gemini NX C18 column (150 × 2.0 mm I.D, 3.0 mm) obtained from Phenomenex (Palo Alto, CA, USA). The mobile phases were solvent A (5 mM ammonium formate and 0.1% formic acid in water) and solvent B (5 mM ammonium formate and 0.1% formic acid in methanol) at a flow rate of 0.25 mL/min. The elution was carried out employing a gradient starting with 10% of B, increasing to 80% up to 1.5 min, and the proportion was kept constant until the fourth min. The ratio was again increased to 90% up to the 10th min. Phase B was then increased to 100% until the 14th min. The time interval between injections was 10 min to return to initial conditions. The injection volume of the samples was 20 µL. The nebulizer, the makeup gas, and the curtain gas were set at 55, 50, and 15 psi, respectively. Furthermore, the capillary temperature was set at 550 °C, and the ion spray voltage was set at 5500 V. Finally, the precursor-to-product ion transitions were m/z 313.3/241.3–228.5 for AFB_1_ [48].

### 4.11. Statistical Analyses

The assays were performed in triplicate (*n* = 9). Analysis of variance (ANOVA) was followed by Tukey’s test using the software GraphPad Prism 5. The results obtained are expressed as mean ± standard deviation. Statistical differences were considered significant if *p* ≤ 0.05.

## Figures and Tables

**Table 1:** Minimum Inhibitory Concentration and minimum fungicidal concentration obtained using allyl isothiocyanate and freeze-dried yellow mustard flour extract against *Aspergillus flavus* ISPA 8111.

Minimum Inhibitory Concentration/Minimum Fungicidal Concentration				
Fungi	Compounds			
	AITC (mg/L)		YMF-E (mg/L)	
	MIC	MFC	MIC	MFC
*Aspergillus flavus*	7.90	31.61	390	3130

MFC: minimum fungicidal concentration; MIC: Minimum inhibitory concentration; AITC: allyl isothiocyanate; YMF-E: freeze-dried yellow mustard flour extract (*n* = 8).

**Table 2:** In vitro antifungal activity of oriental mustard flour antifungal device based on hydroxyethyl-cellulose (H-OMF) against *Aspergillus flavus* ISPA 8111.

Mycelial Growth (mm of Diameter) after Fumigation by H-OMF			
Treatment	Days		
	3	5	7
Control	25 ± 1.2 ^A^	50 ± 2.5 ^A^	50 ± 5.5 ^A^
30 mg/L	ND	ND	ND
25 mg/L	5 ± 1.3 ^B^	25 ± 3.4 ^B^	37 ± 5.1 ^B^
12.5 mg/L	9 ± 0.8 ^C^	34 ± 2.2 ^C^	38 ± 4.2 ^B^
6.2 mg/L	9 ± 1.1 ^C^	48 ± 1.6 ^A^	50 ± 6.1 ^A^

OMF: oriental mustard flour; ND: the mycelial growth was not detected; different capital letters represent statistical differences (*p* ≤ 0.05) (*n* = 9).

**Table 3:** The residual population of *Aspergillus flavus* ISPA 8111 in almonds treated with allyl isothiocyanate, the antifungal device of hydroxyethyl-cellulose and oriental mustard flour, and freeze-dried mustard flour extract.

	Fungal Population in log CFU/g (Mean ± SD)			
Treatment	Concentration	Days		
		0	7	15
Control	-	5.11 ± 0.26 ^A^	9.24 ± 0.34 ^A^	10.52 ± 0.08 ^A^
AITC	5.07 mg/L	5.10 ± 0.16 ^A^	≤1.22 ± 0.00 ^B^	≤1.22 ± 0.00 ^B^
	10.13 mg/L	5.10 ± 0.11 ^A^	≤1.22 ± 0.00 ^B^	≤1.22 ± 0.00 ^B^
	20.26 mg/L	5.70 ± 0.51 ^A^	≤1.22 ± 0.00 ^B^	≤1.22 ± 0.00 ^B^
H-OMF	2000 mg/L	5.90 ± 0.20 ^A^	≤1.22 ± 0.00 ^B^	≤1.22 ± 0.00 ^B^
	4000 mg/L	5.80 ± 0.34 ^A^	≤1.22 ± 0.00 ^B^	≤1.22 ± 0.00 ^B^
YMF-E	100 g/L	5.60 ± 0.27 ^A^	8.91 ± 0.29 ^A^	10.31 ± 0.04 ^A^
	160 g/L	5.40 ± 0.53 ^A^	9.96 ± 0.10 ^A^	10.68 ± 0.16 ^A^
	200 g/L	5.70 ± 0.21 ^A^	8.96 ± 0.17 ^A^	10.15 ± 0.12 ^A^

AITC: allyl isothiocyanate; H-OMF: hydroxyethyl-cellulose device with oriental mustard flour; YMF-E: yellow mustard flour extract. Different capital letters represent statistical differences among treatments (*p* ≤ 0.05). (*n* = 18).

**Table 4:** Effect of allyl isothiocyanate, the antifungal device of hydroxyethyl-cellulose and oriental mustard flour, and yellow mustard flour extract treatment on aflatoxin B_1_ (AFB_1_) production by *Aspergillus flavus* in almonds.

	The Concentration of AFB_1_ in µg/kg (Mean ± SD)		
Treatment	Concentration	Days	
		7	15
Control	-	36.22 ± 4.21 ^A^	71.67 ± 2.84 ^A^
AITC	5.07 mg/L	≤LOD ^C^	≤LOD ^C^
	10.13 mg/L	≤LOD ^C^	≤LOD ^C^
	20.26 mg/L	≤LOD ^C^	≤LOD ^C^
H-OMF	2000 mg/L	≤LOD ^C^	≤LOD ^C^
	4000 mg/L	≤LOD ^C^	≤LOD ^C^
YMF-E	100 g/L	267.38 ± 43.09 ^B^	276.43 ± 186.83 ^B^
	160 g/L	314.13 ± 131.49 ^B^	364.41 ± 77.78 ^B^
	200 g/L	395.21 ± 143.14 ^B^	575.90 ± 169.61 ^B^

AITC: allyl isothiocyanate; H-OMF: antifungal device of hydroxyethyl-cellulose with oriental mustard flour; YMF-E: yellow mustard flour extract; LOD: limit of detection was 0.30 µg/kg; A, B, and C: capital letters represent statistical differences among treatments at the same time point (*p* ≤ 0.05). The samples were previously analysed for the presence of AFB_1_ (*n* = 9).

## Data Availability

The data presented in this study are available in this article.
